# Unusual Brachial Plexus Injury Caused by a Personal Watercraft Fall

**DOI:** 10.7759/cureus.98249

**Published:** 2025-12-01

**Authors:** Orlando De Jesus, Joel Pellot

**Affiliations:** 1 Neurosurgery, University of Puerto Rico, Medical Sciences Campus, San Juan, PRI

**Keywords:** brachial plexus, injury, jet ski, nerve root, ocean, personal watercraft, traction

## Abstract

Brachial plexus injuries after personal watercraft accidents have been rarely reported. We present a 38-year-old female who was riding a personal watercraft when she was struck by an ocean wave and ejected backwards. The motor exam demonstrated bilateral upper extremity weakness. Brachial plexus MRI revealed left C4-C5 and C5-C6 nerve root sleeve tears, with possible similar contralateral injuries. The patient was observed for two days without clinical improvement and was sent home with an appointment to the peripheral nerve clinic and evaluation with a physiatrist. She completed six weeks of physical therapy, achieving substantial improvement, and recovered completely six months later. Traction of the brachial plexus can occur when an individual hangs onto the handlebars with the involved arm while being ejected from a personal watercraft. The expulsion causes a forceful separation motion of the shoulder with respect to the arm or the head with respect to the neck.

## Introduction

Among trauma patients, approximately 1% suffer brachial plexus injuries, most commonly secondary to motorcycle and snowmobile accidents [1]. However, the incidence of injury with motorcycle or snowmobile accidents approaches 5% [1]. The injury to the brachial plexus can be complete or only involve the upper or lower plexus, or a combination of both. Complete lesions have a prevalence of 53%, followed by upper plexus lesions at 39%, and lower plexus injuries at 6% [2]. Several mechanisms for traumatic injury to the brachial plexus have been described, usually involving a forceful stretch of the arm concurrently with extreme motions of the body [3,4]. MRI is the cornerstone imaging evaluation for injuries to the brachial plexus [5]. MRI helps diagnose the injury and accurately identify the site of injury [5,6]. Injuries related to personal watercraft (PWC) accidents have long been recognized due to their increased use [7].

Brachial plexus injuries after PWC accidents have been rarely reported. To the best of our knowledge, only one prior case has been reported in the literature. Sandri et al. described a patient with an injury to the brachial plexus after falling from a high-speed jet ski secondary to the fracture of both first ribs after the high-impact dive [8]. We present a patient who was riding a PWC when she was struck by an ocean wave and ejected backwards, causing injury to both brachial plexuses.

## Case presentation

A 38-year-old female was riding a PWC when she was struck by an ocean wave and ejected forcefully backwards. She remembered holding onto the handlebars as she initially fell. In the water, she immediately started complaining of severe cervical pain extending to her lower back, associated with numbness and tingling in her four extremities. The physical examination revealed an alert and oriented patient with intact deep pain and light touch sensation in all four extremities. The motor exam demonstrated bilateral upper extremity weakness. The hand grip and the triceps muscle were 4/5 bilaterally. Deltoid, biceps, and brachioradialis muscles were 1/5 on the left and 2/5 on the right. Lower extremities showed no weakness. She complained of left elbow dysesthetic pain.

**Figure 1:**
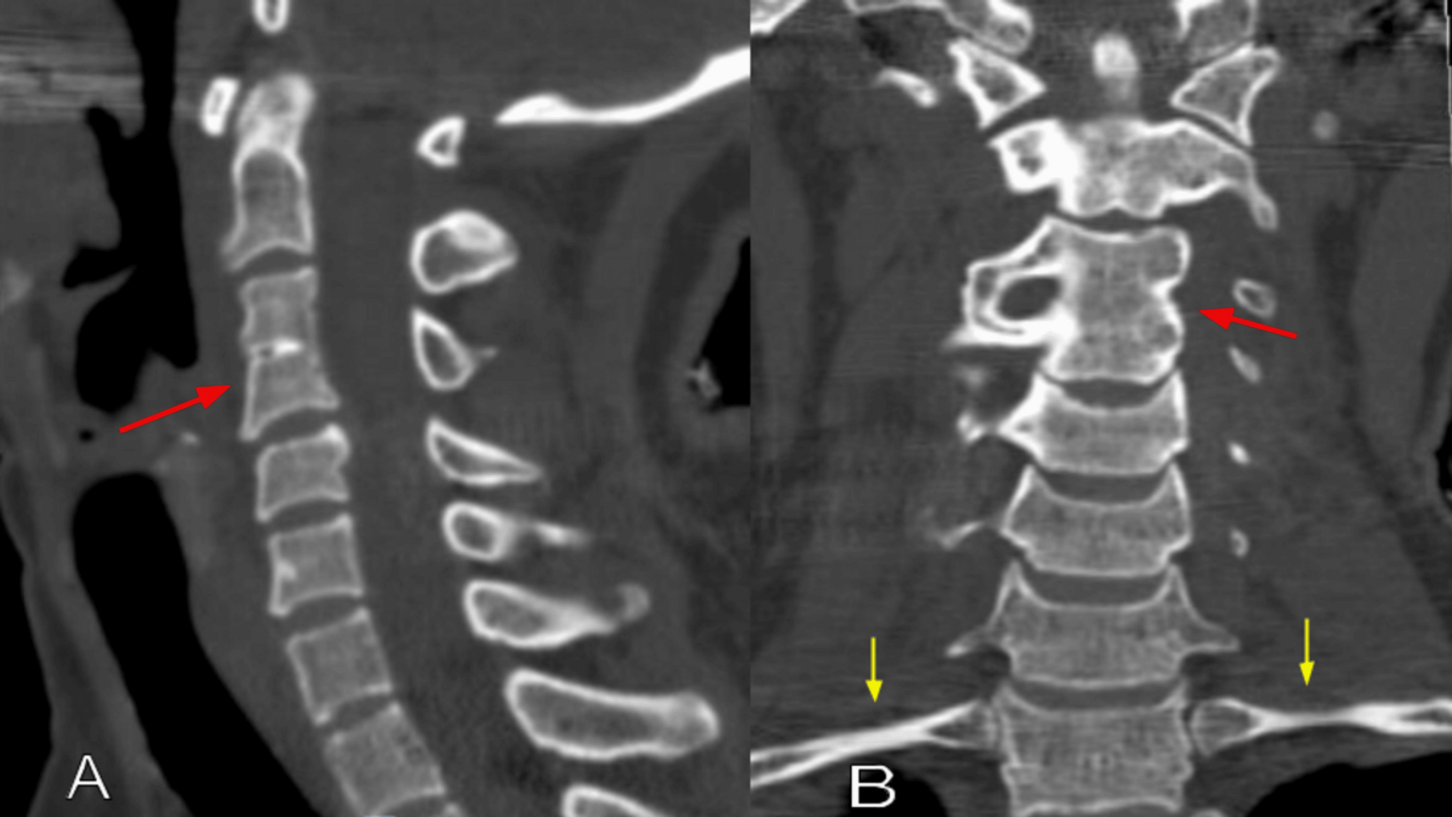
Cervical CT scan bone window (A) sagittal and (B) coronal images showing a C3-C4 vertebral body fusion (red arrows), with intact first ribs (yellow arrows).

**Figure 2:**
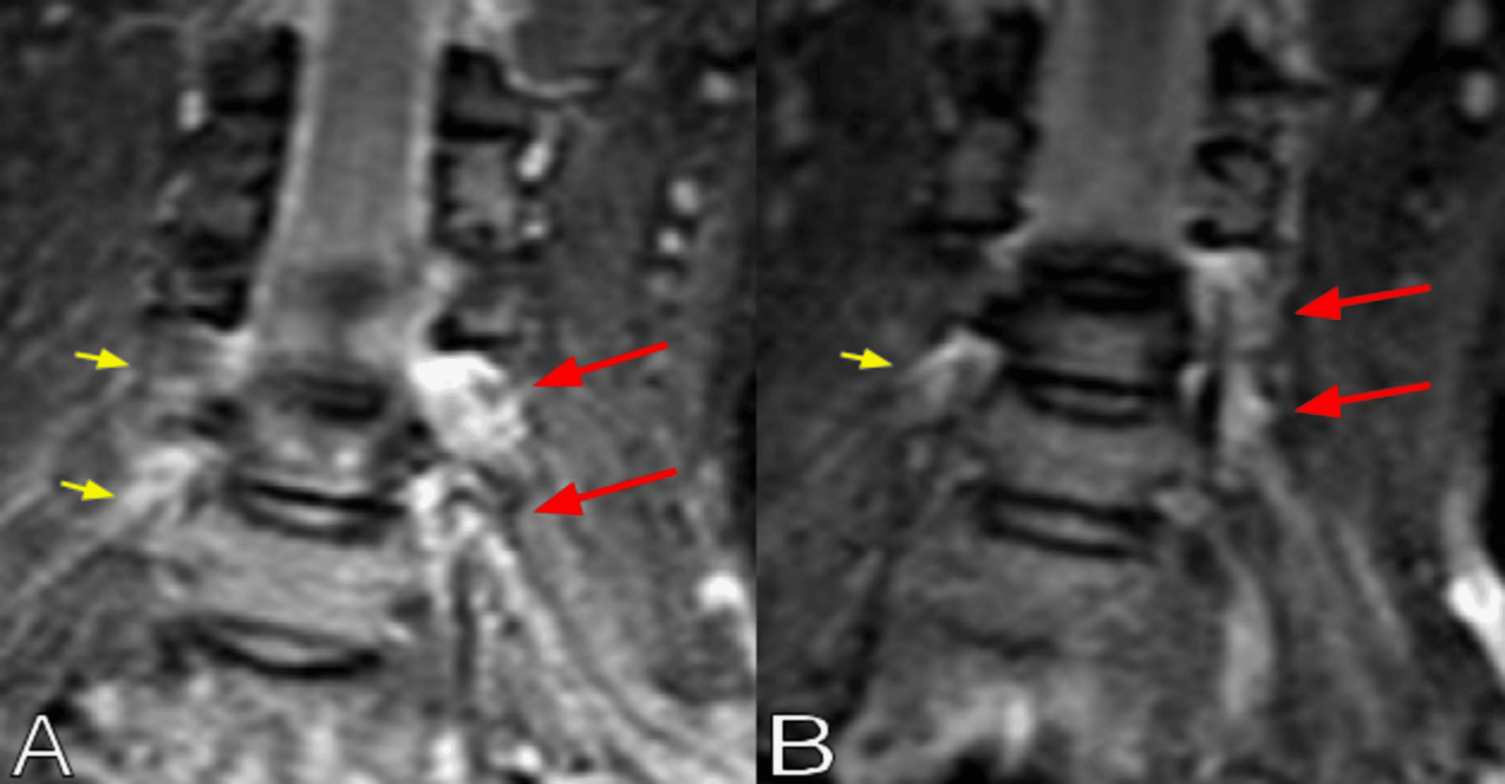
Brachial plexus coronal MRI short tau inversion-recovery (STIR) images showing (A and B) left C4-C5 and C5-C6 nerve root sleeve tears (red arrows), with possible similar contralateral injuries (yellow arrows).

**Figure 3:**
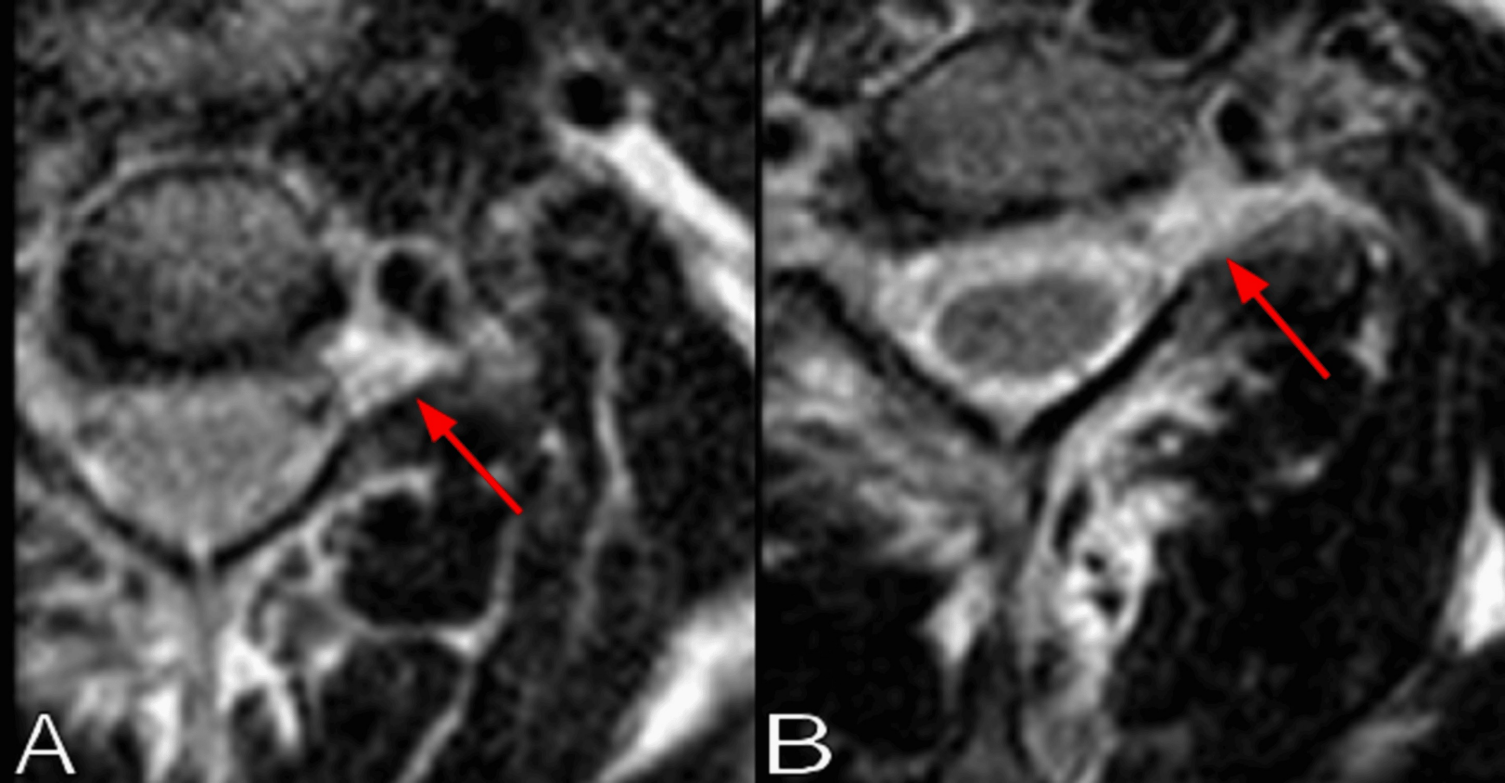
Brachial plexus axial MRI T2-weighted images showing (A) left C4-C5 and (B) left C5-C6 nerve root sleeve tears (red arrows).

A chest radiograph showed no rib fractures. The cervical CT scan showed an incidental C3-C4 vertebral body fusion, but no fractures or dislocations (Figure 1). Brachial plexus MRI revealed left C4-C5 and C5-C6 nerve root sleeve tears, with probable similar contralateral injuries (Figure 2). Typical injuries were noted on the axial T2-weighted images, displaying increased signal at the involved left nerve roots (Figure 3). The patient was observed for two days without clinical improvement. She was sent home with a follow-up appointment at the peripheral nerve clinic and evaluation with a physiatrist. Electromyography and nerve conduction studies were performed three weeks after the injury, demonstrating early recovery in the affected muscles, with the recorded motor unit action potentials approaching normal values. She completed six weeks of physical therapy, showing rapid improvement. The physical treatment included therapeutic exercises to increase muscle strength and range of motion, as well as neuromuscular re-education. Six months after the accident, she had recovered completely, showing 5/5 in all upper extremity muscles. At this time, she was able to perform all daily activities at the pre-injury level, including washing her hair, applying makeup, taking off a shirt, reaching for an item above shoulder level, and opening containers, as measured by the patient-specific functional scale.

## Discussion

PWC's technology has advanced significantly, with current models achieving over 340 horsepower and reaching top speeds of 80 mph [9,10]. These remarkable advancements also raise concerns about the potential for severe injuries. Most PWC injuries occur secondary to a collision with another watercraft or a fixed object, with a smaller portion resulting from forceful ejection, handlebar injury, axial loading, or hydrostatic jet injury [11]. Spinal injuries account for approximately 18-30% of all injuries sustained [9,10]. Handlebar straddle injuries most commonly affect internal organs or the pelvic region when the PWC collides with a fixed object. However, if the rider holds onto the handlebars while being ejected, there is a risk of brachial plexus injury due to the forceful pull of the arm resulting from the forward movement of the PWC while the body is being ejected backwards. The case reported by Sandri et al. had a similar accident mechanism as our patient after falling from a high-speed jet ski; however, the injury to the brachial plexus was provoked by the bruising action of the life vest, which fractured both first ribs after a high-impact dive [8]. Other authors have reported brachial plexus injuries secondary to a first rib fracture, shoulder dislocation, or rotator cuff tear following motorcycle or motor vehicle accidents [12-14]. However, our patient had no associated first rib fracture, shoulder dislocation, or tears, which could have caused the brachial plexus injury.

Damage to the brachial plexus can occur due to nerve avulsion, rupture, stretch, or a tear in the epidural sac [4]. The nerve roots of C5 and C6 are less commonly avulsed compared to the nerve roots of C7 through T1, as they have strong fascial attachments at the spine [3,4]. Tears or avulsion of the mid-cervical nerve roots occur due to distraction, which can happen when a rider falls, having their head violently flexed to the opposite side [15]. MRI of traumatic brachial plexus injuries is a reliable tool with high sensitivity and specificity, showing increased signal on T2-weighted or short tau inversion-recovery (STIR) sequences, best assessed on coronal sections along the plexus course [6,15]. In our patient, the prompt and complete recovery of function suggested that the injury involved only a tear of the epidural sac of the root sleeve, with mild root stretching but without avulsion.

## Conclusions

Traction of the brachial plexus can occur when an individual hangs onto the PWC handlebars with the involved arm while being ejected. The expulsion causes a forceful separation motion of the shoulder with respect to the arm or the head with respect to the neck. This case exemplifies that an extreme pull motion of the arm can cause an injury to the brachial plexus despite the absence of a shoulder dislocation or a first rib fracture.
